# Narrative Review of Neurologic Complications in Adults on ECMO: Prevalence, Risks, Outcomes, and Prevention Strategies

**DOI:** 10.3389/fmed.2021.713333

**Published:** 2021-09-29

**Authors:** Hongling Zhang, Jiqian Xu, Xiaobo Yang, Xiaojing Zou, Huaqing Shu, Zhengdong Liu, You Shang

**Affiliations:** ^1^Department of Intensive Care Unit, Affiliated Liu'an Hospital, Anhui Medical University, Liu'an, China; ^2^Department of Critical Care Medicine, Union Hospital, Tongji Medical College, Huazhong University of Science and Technology, Wuhan, China

**Keywords:** ECMO, neurologic complications, neurologic monitoring, multimodal, strategy

## Abstract

Extracorporeal membrane oxygenation (ECMO), a life-saving technique for patients with severe respiratory and cardiac diseases, is being increasingly utilized worldwide, particularly during the coronavirus disease 2019(COVID-19) pandemic, and there has been a sharp increase in the implementation of ECMO. However, due to the presence of various complications, the survival rate of patients undergoing ECMO remains low. Among the complications, the neurologic morbidity significantly associated with venoarterial and venovenous ECMO has received increasing attention. Generally, failure to recognize neurologic injury in time is reportedly associated with poor outcomes in patients on ECMO. Currently, multimodal monitoring is increasingly utilized in patients with devastating neurologic injuries and has been advocated as an important approach for early diagnosis. Here, we highlight the prevalence and outcomes, risk factors, current monitoring technologies, prevention, and treatment of neurologic complications in adult patients on ECMO. We believe that an improved understanding of neurologic complications presumably offers promising therapeutic solutions to prevent and treat neurologic morbidity.

## Introduction

Extracorporeal membrane oxygenation (ECMO) is an increasingly utilized technique for patients with refractory respiratory and cardiac diseases. Depending on the patient's condition and disease progression, numerous types of ECMO configurations are available. The two most common modes are venoarterial (VA) and venovenous (VV) ECMO (1). ECMO drains venous blood using a centrifugal pump to allow extracirculatory gas exchange (oxygenation and carbon dioxide removal) through an artificial membrane; the oxygenated blood then returns to the circulation through the large arteries (VA ECMO) or through a central vein (VV ECMO). This technique has demonstrated remarkably increasing implementation, as it is consistently utilized as a bridge to recovery and can improve outcomes. Among the complications that can develop during ECMO support, those affecting the nervous system are common and associated with poor survival. In critically ill patients, especially those who received ECMO, median in-hospital mortality is higher in patients with neurologic injury than in those without (2–4). This article reviews the prevalence and outcomes, risk factors, and clinical strategies for neurologic complications in patients on ECMO.

## Neurologic Complications of Patients on ECMO

### Short-Term Complications

Short-term neurologic complications, which usually occur during or immediately after ECMO, mainly consist of intracranial hemorrhage (ICH), acute ischemic stroke (AIS), seizure, hypoxic-ischemic brain injury (HIBI), and brain death. The prevalence and mortality of neurologic complications vary across different adult ECMO centers (Table 1). However, the true prevalence may be underestimated; some patients with neurologic complications may have been diagnosed with multiple organ failure and thus may have died without undergoing neuroimaging, while in other instances, a lack of routine neurologic monitoring and standardized diagnostic criteria may have masked the prevalence of neurologic complications. Because research regarding neurologic injury in hybrid ECMO modes is lacking, this paper specifically focuses on neurologic complications in patients on VA and VV ECMO.

**Table 1:** Studies investigating the prevalence and mortality of neurologic complications in adult patients on ECMO.

**References**	**Number of enrolled patients**	**Prevalence% (mortality%)**							
		**ECMO**	**VA ECMO**	**VV ECMO**	**ICH**	**AIS**	**Seizures**	**HIBI**	**BD**
Sutter et al. (2)	N/A	13.0 (83.0)	15.0	10.0	5.0 (96.0)	7.0 (83.0)	2.0 (40.0)	N/A	N/A
Shoskes et al. (5)	16,063 (VA 8211, VV 7842)	16.0 (47.0)	19.0 (52.0)	10.0 (36.0)	7.0	7.0	1.0	8.0	N/A
Chapman et al. (6)	412 (VA 244, VV 131, Other type 37)	13.3 (65.0)	18.0	4.5	3.4	7.0	N/A	3.6	N/A
Lorusso et al. (7)	4,522 (all VA)	N/A	15.1 (90.0)	N/A	1.8	3.6	1.8	N/A	7.9
Lorusso et al. (8)	4,988 (all VV)	N/A	N/A	7.1 (75.8)	3.6 (79.6)	1.7 (68.2)	1.2 (50.0)	N/A	2.0
*Lockie et al. (9)	250 (all VV)	N/A	N/A	N/A	16.4 (31.7)	N/A	N/A	N/A	N/A
*Hunsicker et al. (10)	444 (all VV)	N/A	N/A	N/A	11.0 (69.4)	N/A	N/A	N/A	N/A
Lorusso et al. (11)	6,834 (all VV)	N/A	N/A	7.2 (76.3)	3.8 (78.5)	1.7 (73.0)	1.2 (54.1)	N/A	N/A

*N/A, not applicable; ECMO, extracorporeal membrane oxygenation; ICH, intracranial hemorrhage; AIS, acute ischemic stroke; HIBI, hypoxic-ischemic brain injury; BD, brain death; VV, venovenous; VA, venoarterial.*

*Study investigated only the incidence of ICH among patients on VV ECMO.

### Neurologic Complications in VA ECMO

VA ECMO is an effective mechanical circulatory support system that rapidly restores systemic perfusion in patients with poor cardiac function (12, 13). Neurologic complications are reported more frequently for patients on VA ECMO than for those on VV ECMO (2, 6, 11). The higher incidence is probably due to the inclusion of patients undergoing extracorporeal cardiopulmonary resuscitation (ECPR). One study showed that almost one-third of patients undergoing ECPR experienced neurologic complications, with brain death being the most frequent complication in the ECPR group (14). In addition, a meta-analysis found that upon excluding studies including an ECPR population, overall neurologic complications were similar between patients on non-ECPR VA ECMO and VV ECMO (5). According to previous research, AIS is the predominant complication in patients on VA ECMO, probably owing to the reduced blood flow in the left side of the heart and thrombosis in the cardiac circuit or cannula (15, 16). Meanwhile, in patients supported with peripheral VA ECMO, a distinctive phenomenon called differential hypoxia or Harlequin syndrome can occur once combined with residual heart function and respiratory failure due to ejection of deoxygenated blood from the left heart to the aorta, which can result in brain and upper body hypoxia relative to the lower body (17–19). Recently, some studies describing brain autopsies for decedents who previously received ECMO showed a high proportion of HIBI in VA ECMO patients (20, 21); hence, we must highlight these important concerns.

### Neurologic Complications in VV ECMO

VV ECMO provides pulmonary support for patients with severe acute refractory and reversible respiratory failure and enables lung-protective ventilation (22). For patients supported with VV ECMO, ICH is the predominant neurologic event and is associated with high mortality (9, 10). The most common types of ICH are subarachnoid hemorrhage and petechial intraparenchymal hemorrhage. Notably, ICH often occurs at an early stage, even immediately after ECMO initiation, without persistent coma or other positive neurologic signs (anisocoria, mydriasis, etc.) (9, 10, 23, 24). Remarkably, one study with severe respiratory failure patients supported using VV ECMO showed that a significant proportion of ICH occurred at admission (within 6 h of ECMO implantation). Neurologic injury at the pre-clinical stage could possibly be detected with a higher and earlier proportion of cranial computed tomography (CT) scans (9). Owing to meticulous anticoagulation therapy and frequent clinical neurologic assessment after prior detection, the above-mentioned study showed a considerable survival among patients on VV ECMO with ICH compared with many other reports (8, 10, 11). Therefore, earlier identification and intervention are associated with survival and good neurologic outcomes.

### Long-Term Complications

During long-term follow-up, survivors treated with ECMO often experienced depression, anxiety, and post-traumatic stress after discharge (25–27). Interestingly, in a prospective observational study with a mean follow-up time of 23 months, some subtle neurologic complications were detected; ECMO patients had an evidently increased patellar tendon reflex and right gastrocnemius tendon reflex, which impaired quality of life and psychological health (28). In addition, a systematic review recently showed that ECMO survivors variably displayed cognitive and psychiatric symptoms more than a year after discharge that reduced their quality of life (29). However, some studies have shown that patients treated with VV ECMO for respiratory failure may have normal cognitive function years after treatment if they were not affected by cerebrovascular lesions (30, 31). Nevertheless, it is important for survivors to undergo neuroimaging at the time of discharge. Furthermore, to improve the quality of life, high-risk patients should be followed up to monitor their neuropsychological condition.

## Risk Factors

Changes in cerebral autoregulation and perfusion and coagulopathy (due to illness or anticoagulation strategy) may increase the risk of neurologic injury in patients on ECMO (24). Importantly, it is difficult to determine whether neurologic injury is related to ECMO support or to the primary illness (severe hypoxemia, shock state, coagulation dysfunction, etc.) and treatment (mechanical ventilation, vasoactive agent administration, etc.). Different studies have revealed a variety of risk factors for neurologic complications in patients on ECMO (2, 6–10, 32, 33) (Table 2), which may be explained by the differences in the ECMO centers in which these studies were conducted, including the device, anticoagulation management, and proportion of cranial CT scans implemented as well as individual patient differences. However, regression analyses cannot demonstrate a direct cause-effect relationship, and more studies are needed to better define the causes of neurologic complications associated with ECMO.

**Table 2:** Risk factors for neurologic complications in adult patients on ECMO in selected studies.

	**Neurologic injury**	**Intracranial hemorrhage**	**Acute ischemic stroke**
Risk factors	Pre-ECMO cardiac arrest Age VA ECMO Post-ECMO hypoglycemia Use of inotropes Renal replacement therapy Hyperbilirubinemia	Pre-ECMO PHPre-ECMO PO_2_Initial decrease in PaCO_2_Duration of mechanical ventilation and ECMOHigh PEEPFemale sexDecreased serum fibrinogenUse of heparinIncreased creatinineRenal replacement therapyThrombocytopeniaGastrointestinal hemorrhage	Pre-ECMO serum lactic acid Pre-ECMO PH Hemolysis Gastrointestinal hemorrhage Disseminated intravascular coagulation

*ECMO, extracorporeal membrane oxygenation; VV, venovenous; VA, venoarterial; PH, potential of hydrogen; PEEP, positive end-expiratory pressure*.

## Clinical Strategies

### Neurologic Monitoring Methods

For patients with neurologic complications on ECMO that are usually associated with poor outcomes, clinical strategies to recognize these complications in a timely manner should be considered. Clinicians and nurses should routinely perform neurologic examinations on patients on ECMO support, including the Glasgow coma scale, pupil examinations (size, shape, equality, reflex to light), and brainstem reflex, tendon reflex, and pathological reflex tests. However, patients undergoing ECMO are always sedated, and positive signs of neurologic injury may be missed despite the implementation of comprehensive clinical examinations. In addition, some patients on ECMO remain comatose after weaning from sedation and neuromuscular blocking agents; in these situations, safe, non-invasive, high-sensitivity and high-specificity monitoring methods should ideally be implemented early. The current monitoring methods for neurologic injury in patients on ECMO are presented in Table 3.

**Table 3:** Current non-invasive clinical technologies for neurologic monitoring in patients on ECMO.

**Monitoring technique**	**Description**
Neurological pupil index (NPi)	• Automatedly assesses pupil size • Neurological deterioration is always associated with a sudden drop in NPi value below 2.8–3.0 • Influenced by ambient light and high concentrations of opioids
Cerebral near-infrared spectroscopy	• Monitors of regional oxygen saturation (rSO_2_) • Decreases from baseline (>25% drop) and a large right-left rSO2 difference (>10%) may predict acute neurologic injury • Affected by ambient light and skin color
Electroencephalography (EEG)	• Assess neurologic status • Continuous EEG can detect non-convulsive seizures or status epilepticus • Occurrence of suppressed EEG background is associated with poor neurological outcome and mortality • Early Standard EEG can be used for prognostication • As the results of EEG depend on technician's experience and expertise, quantitative EEG can be applied in the future studies
Transcranial doppler (TCD) ultrasound	• Measures the pattern of cerebral blood flow, hemodynamic reserve, and microembolic signals • Effectiveness of TCD is uncertain with continuous, non-pulsatile, arterial flow in some patients on venoarterial ECMO • Detects intracranial artery microembolic signals, which may eventually lead to acute ischemic stroke
Biomarkers	• Serum protein biomarkers of cerebral injury, such as NSE, and S100B • Serial measurement can increase diagnostic accuracy • Serum half-life of NSE is long, and can be affected by hemolysis or cancer • S100B can be released from not only brain but also heart, bone and adipose tissue
Neuroimaging	• Cranial CT is the main technology for revealing and excluding acute cerebral injury • MRI is limited due to hardware incompatibility

*ECMO, extracorporeal membrane oxygenation; NSE, neuron-specific enolase; CT, computerized tomography; MRI, magnetic resonance imaging*.

### Neurological Pupil Index

Conventional methods of pupillary evaluation are affected by the examiner's subjectivity. Nevertheless, the neurological pupil index (NPi), an automated pupillary assessment tool that combines multiple quantitative variables, such as minimal and maximal pupil sizes, constriction velocity and latency, to calculate a single index, is a sensitive measure of pupillary reactivity that can be used as an early, non-invasive indicator of increasing intracranial pressure (ICP) (34–36). Neurologic deterioration is associated with a sudden drop in the NPi value below 2.8, regardless of the side of the lesion (37). In addition, the NPi might be a useful parameter for estimating the severity of aneurysmal subarachnoid hemorrhage, in which reduced NPi values are associated with poor clinical outcomes (38). Recently, a single-center study showed that an abnormal NPi <3 from 24 to 72 h following VA ECMO insertion had 100% specificity and 53% sensitivity in predicting 90-day mortality (39). However, several aspects should be considered when the NPi is applied. In critically ill patients, the NPi is affected by ambient light; the use of an opaque rubber cup could reduce the effect of ambient light conditions on quantitative pupillometry (40, 41). The NPi is influenced to a small degree by sedation analgesia, but high concentrations of opioids may unduly affect its value (42). Finally, the pupillary light reflex, when assessed using a pupillometer, is not dependent on eye color (43). Further research on establishing the correlation between NPi and neurologic outcomes in ECMO patients is needed.

### Near-Infrared Spectroscopy

Cerebral near-infrared spectroscopy (NIRS) is widely used as a validated, continuous, and non-invasive method for monitoring variations in regional oxygen saturation (rSO_2_) in patients undergoing ECMO. Cerebral NIRS can function as a first-alert monitor to warn of neurologic complications occurring in patients on both VA and VV ECMO (44). During NIRS monitoring, acute cerebral complications were more frequent in VA ECMO patients with the lowest rSO_2_ value (optimum cutoff value 52%, sensitivity 71% and specificity 72%) or a large right–left rSO_2_ difference (>10%) (45). A single-center prospective cohort study of VA ECMO patients found that all patients with acute brain injury experienced rSO_2_ desaturation during ECMO, indicating that normal rSO_2_ values are extremely sensitive in predicting patients who will not suffer from neurologic injury (46). More recently, one study showed that cerebral NIRS was a useful, real-time bedside neuromonitoring monitor, demonstrating that a drop in rSO_2_ > 25% from baseline (with a sensitivity of 86% and a specificity of 55%) predicted acute neurologic injury in patients on VA ECMO (47). Furthermore, cerebral NIRS has also been shown to correlate directly with cardiac output values. Cerebral tissue oxygenation is lowered during episodes of atrial fibrillation and ventricular fibrillation in patients on VA ECMO, indicating that cerebral oximetry readings could adequately reflect hemodynamic instability, as it is associated with a relative decrease in cerebral perfusion (48). However, despite its obvious benefits, NIRS can also be affected by irrelevant factors such as skin color and ambient light. Compared with minimum rSO_2_ values, decreases in rSO_2_ values from baseline may better predict the occurrence of acute brain injury.

### Electroencephalography

EEG is a non-invasive technique to assess neurologic status in critically ill patients (49). Subclinical or electrographic seizures are common in critically ill populations, and continuous EEG monitoring can be used to detect pathologic changes such as non-convulsive seizures or status epilepticus. Moreover, the discovery of epileptic potential may change the management of antiepileptic drugs (50, 51). Table 1 shows that the prevalence of seizures in patients on ECMO is low, mainly due to the use of anesthetics and a lack of EEG monitoring. A multicenter retrospective cohort study demonstrated that non-convulsive seizures or status epilepticus are common in patients with COVID-19 undergoing clinically indicated continuous EEG, that electrographic seizures were an independent predictor of in-hospital mortality and that the presence of non-convulsive status epilepticus prolonged the time of hospitalization (52). Recently, some investigations have highlighted the importance of EEG in adult ECMO patients. One retrospective analysis on the background EEG signal of patients undergoing both VA and VV ECMO classified it into four categories: mild/moderate encephalopathy, severe encephalopathy, burst suppression and suppressed background (53). The researchers observed that 38% of patients presented with a severe EEG background abnormality, 15% of whom presented with seizures or periodic discharges. The occurrence of suppressed background EEG signals was significantly associated with both unfavorable neurologic outcomes and hospital mortality. However, the study did not standardize the timing for initiating EEG monitoring in all their patients. Two studies confirmed that background abnormalities on standard EEG at the early phase (1–3 d) were highly predictive of brain injury and poor outcome in patients on VA ECMO (54, 55). Furthermore, the authors also found that a lack of sleep transients on continuous EEG reflects the severity of brain dysfunction and might serve as an additional prognostic marker. However, the results of EEG depend on the experience and expertise of the technicians; quantitative EEG, on the other hand, is automated and may shorten the EEG review time with better sensitivity for neurologic monitoring in ICU patients (56, 57), but the evidence for adult patients on ECMO is insufficient, and further research is required.

### Transcranial Doppler Ultrasound

Transcranial Doppler (TCD) ultrasound is a bedside technique that can be safely and easily performed in the intensive care unit (ICU). TCD ultrasound offers significant diagnostic value for patients supported by ECMO, providing direct measurements of the pattern of cerebral blood flow, hemodynamic reserve, and microembolic signals (MESs). Waveforms in patients supported by VA ECMO were demonstrated as continuous flows without clear systolic peaks (58). Furthermore, TCD ultrasound can be used to calculate mean flow velocities and pulsatility indexes for patients on VA ECMO (59). As we know, patients on VA ECMO with unrecovered cardiac function have continuous and non-pulsatile blood flow, which is absent in VV ECMO. In this situation, still it is difficult to decide whether TCD ultrasound can be used to confirm brain death due to its inability to detect non-pulsatile blood flow.

Early prognostication of brain death is important to avoid the implementation of ineffective therapy. A loss of spectral Doppler signal in the middle cerebral artery may be an indicator of brain death for patients with non-pulsatile arterial flow (60). During VA ECMO, it is necessary to detect a pulsed waveform in patients with spontaneous heart function or those who were treated with an intra-aortic balloon pump (61, 62); under these circumstances, TCD ultrasound can be a reliable test for diagnosing cerebral circulatory arrest.

Currently, TCD ultrasound is the sole technique capable of detecting MESs in the intracranial arteries. MESs may be caused by solids or gases produced during thrombosis within the circuit or cannula during ECMO and are more common in patients on VA ECMO because during VV ECMO, the lung can act as a filter for thrombi (16, 63–66). An observational prospective study in patients treated with ECMO evaluated by TCD ultrasound showed that the percentage of MES-positive patients was significantly different between the two ECMO configurations (81.8% among VA ECMO patients vs. 26.2% among VV ECMO patients). In addition, compared with that in the VV ECMO group, the number of MESs in the VA ECMO group was more substantial (63). More studies are needed in the future to determine the relationship between MESs and brain injury in patients undergoing ECMO, and TCD ultrasound studies may even help to guide therapeutic approaches for optimal anticoagulation strategies to decrease stroke risk.

### Biomarkers

Some serum protein biomarkers of cerebral injury have been studied in small adult ECMO cohorts, such as neuron-specific enolase (NSE) and S100B. The reliability of serum NSE monitoring indicating relevant neurologic injury for patients with VA ECMO after cardiopulmonary resuscitation was confirmed in two previous studies (67, 68). In one study, patients with NSE <100 g/L had an in-hospital mortality rate of 36.4%, while the percentage demonstrating good neurological status was 67.9%. In addition, when NSE peaks were used to predict neurologic injury, the specificity and sensitivity were 0.74 and 0.6 (cutoff value of 100 g/L) and 0.98 and 0.3 (cutoff value of 200 g/L), respectively (67). In another study aiming to increase the diagnostic accuracy of NSE, the authors measured its level after 24, 48, and 72 h using single NSE measurements, serial NSE measurements and their combination, respectively, to predict cerebral outcome. They found that 48-h NSE measurements showed the best presentation for poor cerebral outcome [area under the receiver operating characteristic (ROC) curve (AUC) of 0.87; cutoff value of 70 μg/L]; moreover, serial NSE measurements in particular demonstrated high specificity (68). When using NSE as a neurological marker, it is essential to consider potential interfering factors, such as hemolysis or cancer. There is an obvious correlation between NSE and hemolysis markers (69), which is relevant during ECMO support, even after a short time of cardiopulmonary bypass.

Several studies instead investigated S100B over NSE as a monitoring biomarker owing to its shorter effective serum half-life. S100B levels were higher in patients with neurologic complications than in those without complications, indicating that the measurement of serum S100B levels could help to provide an early indication of neurologic complications in deeply sedated patients undergoing both VA and VV ECMO (70). In addition, S100B measurements at 40 and 140 h following both VA and VV ECMO initiation showed the best predictions for intracranial lesions (AUC 0.81; cutoff value 0.69 μg/L) (71). However, S100B is not a brain-specific biomarker, as it can be released from the heart, bone, and adipose tissue (70, 72). The combination of various biomarker measurements can result in an increased diagnostic accuracy in predicting brain injury.

### Neuroimaging

Cranial CT is the primary imaging method used to reveal or exclude acute intracranial complications in ECMO patients. CT scans can be used to detect ICH with high sensitivity; however, they are unable to demonstrate dynamic changes and have low sensitivity for small or brainstem infarcts. A study (9) showed that with increased use of CT scans in patients on VV ECMO, clinicians should be able to identify more neurologic injuries earlier and therefore direct changes in therapy, such as anticoagulation, to reduce associated morbidity and mortality. However, the benefits from cranial CT should be balanced against the risks associated with the transportation of ECMO patients. Both VA and VV ECMO are associated with significant neurologic morbidity; before or immediately after ECMO initiation, patients should thus be examined by cranial CT scans if they can be transferred. During ECMO support, cranial CT scans are needed when there exist suspected acute neurologic injuries. Patients undergoing ECMO are unable to undergo magnetic resonance imaging (MRI) due to hardware incompatibility. A small study described MRI images of 8 patients who survived after ECMO cannulation, which found that all MRI images were abnormal with punctate ischemic infarct being the most common finding that may be associated with MESs detected by TCD ultrasound (73).

## Ways to Improve Neurologic Outcomes

### Recognition

**Figure 1:**
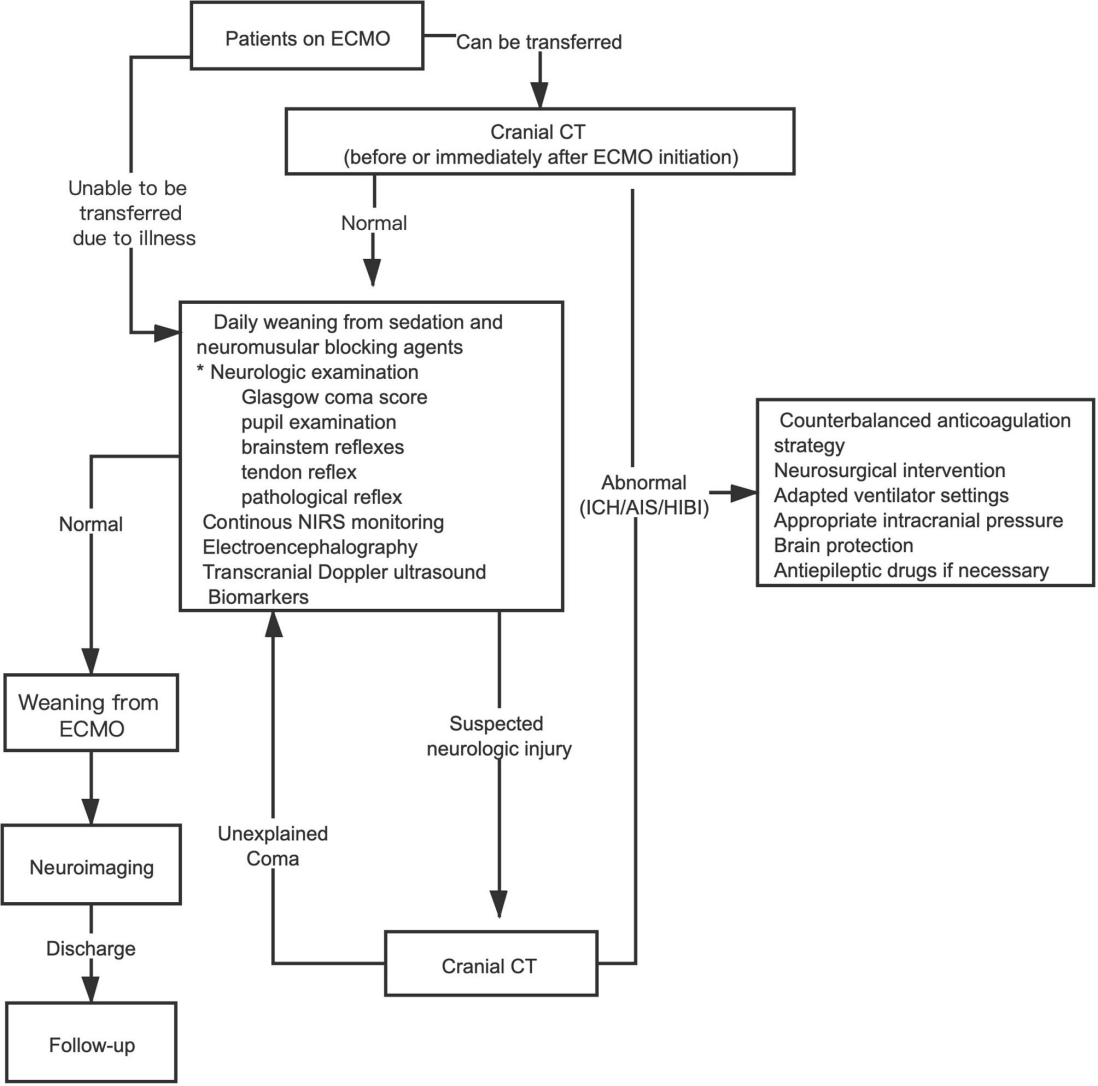
Framework for an earlier recognition and intervention of neurologic complications in patients on ECMO. *Awake ECMO (non-intubated, spontaneously breathing) patients only need detailed neurologic examination. ECMO, extracorporeal membrane oxygenation; CT, computerized tomography; ICH, intracranial hemorrhage; AIS, acute ischemic stroke; HIBI, hypoxic-ischemic brain injury; NIRS, near-infrared spectroscopy.

Patients on ECMO require daily weaning from sedation and neuromuscular blockers and detailed clinical neurologic examinations to identify positive neurologic signs. However, for sedated and comatose patients on ECMO, an optimal monitoring method is still unclear; therefore, the use of multimodal monitoring is required. Electrographic activity and cerebral autoregulation are different in patients with or without neurologic injury. Recently, several studies with small sample sizes described the feasibility of using multimodal non-invasive technology at beside for patients on both VA and VV ECMO (74–76). Based on these studies, we propose a framework for neurologic care in patients on ECMO (Figure 1). Recently, some ECMO centers began using ECMO with non-intubated, spontaneous breathing, called awake ECMO; in addition to reducing complications associated with sedation and invasive mechanical ventilation, it is more likely that neurologic injury can be detected in the early stage using this technique (77–79). For the early diagnosis of neurologic complications, awake ECMO should be used at a suitable opportunity in future procedures.

### Prevention

Some critically ill patients may present with coagulopathy before ECMO initiation, which may increase the risk of ICH. In addition, physicians may withhold anticoagulation when active bleeding (such as gastrointestinal hemorrhage) is present, which may increase the risk of AIS. Therefore, during ECMO support, meticulous anticoagulation strategies should be implemented. PaCO_2_ is an attractive target for potentially improving cerebral perfusion and outcomes. Several studies have revealed a U-shaped relationship between PaCO_2_ during post-resuscitation and both neurologic outcomes and patient survival, meaning that both hypercapnia and hypocapnia are associated with worse outcomes (80, 81). A U-shaped relationship was also found between PaCO_2_ before ECMO initiation and in-hospital mortality (82). Recently, some studies found that acute and sudden decreases in PaCO_2_ over the first 24 h were correlated with a high risk of neurologic complications in patients regardless of the VV or VA ECMO mode (10, 33, 82, 83). The following formula can be used to calculate the relative change in PaCO_2_ during the first 24 h:

RelΔPaCO_2_ = (post-ECMO) PaCO_2_ – (pre-ECMO) PaCO_2_/(pre-ECMO) PaCO_2_

A RelΔPaCO_2_ < -50% is significantly associated with the incidence of neurologic complications (83). Therefore, when ECMO is active, clinicians should monitor arterial blood gases frequently or end-tidal carbon dioxide continuously and start a low sweep gas flow that progressively increases over time to avoid excessively rapid changes in PaCO_2_. When patients with poor cardiac function received VA ECMO, the brain is oxygenated by extracirculatory gas exchange. For patients on both VA and VV ECMO, early hyperoxia (within 24 h after ECMO onset) is significantly associated with higher mortality (84, 85). Furthermore, early hyperoxia increases the risk of acute brain injury and unfavorable neurologic outcomes (84). However, the most appropriate strategy and correction target for PaO_2_ and PaCO_2_ in patients undergoing ECMO remain uncertain and need to be further studied.

### Treatment

Disappointingly, few studies have investigated counterbalancing anticoagulation strategies once ICH occurs during ECMO support. Ongoing anticoagulation may increase the size of any hemorrhage, while discontinuation may induce thrombotic events. We now know that we can reduce the use of anticoagulants and set a lower target activated partial thromboplastin time value with detailed, frequent neurologic examinations and follow-up cranial CT scans. Once AIS occurs during ECMO, the use and timing of mechanical thrombectomy need to be considered. While the literature is limited, one case report described two patients on VA ECMO with AIS treated with mechanical thrombectomy with good neurologic outcome, confirming the feasibility of this treatment (86). When patients on VA ECMO experience differential hypoxia, adapted ventilator settings or hybrid modes are needed to minimize the risk of cerebral desaturation. In addition, regardless of the type of neurologic injury, patients need appropriate intracranial pressure and a series of brain protection strategies.

## Conclusion

With the rapidly increasing use of ECMO, neurologic injuries have become a matter of greater concern, as they are related to increased ICU and hospital stays, morbidity, mortality, and even long-term quality of life. Therefore, to improve the outcomes of ECMO patients, we should strive to prevent or recognize neurologic complications earlier.

## Author Contributions

HZ, JX, ZL, and YS designed the review. HZ and JX wrote the manuscript with supervision of YS. All authors critically revised the manuscript and approved it for publication.

## Conflict of Interest

The authors declare that the research was conducted in the absence of any commercial or financial relationships that could be construed as a potential conflict of interest.

## Publisher's Note

All claims expressed in this article are solely those of the authors and do not necessarily represent those of their affiliated organizations, or those of the publisher, the editors and the reviewers. Any product that may be evaluated in this article, or claim that may be made by its manufacturer, is not guaranteed or endorsed by the publisher.
